# Integrating community-based health promotion programs and primary care: a mixed methods analysis of feasibility

**DOI:** 10.1186/s12913-018-2866-7

**Published:** 2018-01-31

**Authors:** Aaron L. Leppin, Karen Schaepe, Jason Egginton, Sara Dick, Megan Branda, Lori Christiansen, Nicole M. Burow, Charlene Gaw, Victor M. Montori

**Affiliations:** 1 Knowledge and Evaluation Research Unit, Mayo Clinic, 200 First Street SW, Rochester, MN 55905 USA; 2 Division of Health Care and Policy Research, Mayo Clinic, 200 First Street SW, Rochester, MN 55905 USA; 3 Southeastern Minnesota Area Agency on Aging, 2720 Superior Drive NW, Suite 102, Rochester, MN 55901 USA; 4 Dan Abraham Healthy Living Center, Mayo Clinic, 200 First Street SW, Rochester, MN 55905 USA; 5 Mayo Clinic School of Medicine, Mayo Clinic, 200 First Street SW, Rochester, MN 55905 USA

**Keywords:** CBPR, Community-based participatory research, Mixed methods, Implementation, Evidence-based programs, Chronic disease self-management program, Clinic-community linkages, Primary care, Chronic disease management

## Abstract

**Background:**

Implementation of evidence-based programs (EBPs) for disease self-management and prevention is a policy priority. It is challenging to implement EBPs offered in community settings and to integrate them with healthcare. We sought to understand, categorize, and richly describe key challenges and opportunities related to integrating EBPs into routine primary care practice in the United States.

**Methods:**

As part of a parent, participatory action research project, we conducted a mixed methods evaluation guided by the PRECEDE implementation planning model in an 11-county region of Southeast Minnesota. Our community-partnered research team interviewed and surveyed 15 and 190 primary care clinicians and 15 and 88 non-clinician stakeholders, respectively. We coded interviews according to pre-defined PRECEDE factors and by participant type and searched for emerging themes. We then categorized survey items—before looking at participant responses—according to their ability to generate further evidence supporting the PRECEDE factors and emerging themes. We statistically summarized data within and across responder groups. When consistent, we merged these with qualitative insight.

**Results:**

The themes we found, “Two Systems, Two Worlds,” “Not My Job,” and “Seeing is Believing,” highlighted the disparate nature of prescribed activities that different stakeholders do to contribute to health. For instance, primary care clinicians felt pressured to focus on activities of diagnosis and treatment and did not imagine ways in which EBPs could contribute to either. Quantitative analyses supported aspects of all three themes, highlighting clinicians’ limited trust in community-placed activities, and the need for tailored education and system and policy-level changes to support their integration with primary care.

**Conclusions:**

Primary care and community-based programs exist in disconnected worlds. Without urgent and intentional efforts to bridge well-care and sick-care, interventions that support people’s efforts to be and stay well in their communities will remain outside of—if not at odds with—healthcare.

**Electronic supplementary material:**

The online version of this article (10.1186/s12913-018-2866-7) contains supplementary material, which is available to authorized users.

## Background

Half of all American adults have at least one chronic condition; one in 4 live with the burdens of multiple chronic conditions [1]. When individuals with chronic illness are skilled at self-managing their health, they can persevere and thrive [2, 3]. Unfortunately, many people lack the capacity, resources, and/or skills to live well with chronic disease [4–6]. Healthcare organizations desire to support patients in their journeys toward better health [7], but their usual isolation from the communities in which patients live limits their impact [8, 9]. Interventions that occur in the places where people live their lives may better support the needs of patients with chronic illness. As such, multiple stakeholders are advocating for the implementation of evidence-based health promotion programs (EBPs) in the community [10–12].

EBPs are a collection of interventions that are developed and evaluated by researchers for the purpose of enhancing patient capacity to prevent and self-manage chronic health conditions. Typically, EBPs follow a scripted curriculum and are designed to be facilitated with high fidelity by trained lay leaders in community settings. Many have been shown in large trials to improve health outcomes and reduce costs [13–20].

The Chronic Disease Self-Management Program (CDSMP) is a key example [13]. The CDSMP is a group-based workshop delivered over 6 weekly sessions by two trained peer leaders and for a group of 8–15 participants with any chronic physical or mental health condition. Federally-sponsored evaluations of the program have proven its value [14, 21] and its widespread implementation is now a key priority of the United States Department of Health and Human Services (DHHS), the Agency for Healthcare Research and Quality (AHRQ), the Centers for Disease Control (CDC), and the National Council on Aging, among others [10, 12]. Arguably, half of the American adult population could benefit from participating in the CDSMP [21].

Because the CDSMP and other EBPs are offered outside of the traditional healthcare system and delivered by non-traditional healthcare workers, they present unique challenges for implementation [22, 23]. We sought to understand, categorize, and richly describe key challenges and opportunities related to integrating EBPs into routine primary care practice in the United States, using the CDSMP as a test case.

## Methods

### Overview and setting

**Fig. 1:**
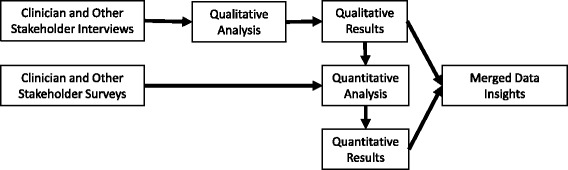
Study overview

The present study is a mixed methods, community engaged research evaluation informed by the Predisposing, Reinforcing, and Enabling Constructs in Environmental Diagnosis and Evaluation (PRECEDE) implementation planning model [24, 25]. It represents the preliminary research component of a parent, participatory action research [26] project focused on EBP implementation in an 11 county region of Southeast Minnesota. The parent project, which was designed to capitalize on the “partnership synergy” [27] of participatory implementation research [28], began when community stakeholders expressed a desire to implement the CDSMP in an effort to meet regional health needs. The community stakeholders were particularly interested in (1.) exploring barriers to clinical integration and (2.) developing strategies to promote referrals from healthcare to the community-based program. We used the PRECEDE planning model to guide these efforts because it has been used successfully in many similar projects [25, 29]. Specifically, we oriented the model to assist—through a coded analysis of interviews and surveys—in identifying the predisposing, reinforcing, and enabling factors and the administrative and policy issues at multiple levels and across sectors that might be relevant to clinically integrating the CDSMP (see Table 1). We designed the study to include qualitative (interview) and quantitative (survey) components conducted in parallel, analyzed in series, and merged according to pre-defined methods (see Fig. 1). We collected both types of data at the same time and for the same exploratory purpose because this was most efficient. We waited, however, to design and conduct the quantitative analyses until after the qualitative analyses were complete. In this way, we hoped to ensure that the survey items corresponded to the appropriate PRECEDE constructs and that the quantitative data could be used to both strengthen and confirm the qualitative insight. We chose this approach—consistent with best practices for mixed methods research [30]—to gain rich and reliable understanding that would be of practical use in developing implementation strategies that would be both locally useful and broadly applicable [31].

**Table 1:** The PRECEDE framework as conceptualized for this study

PRECEDE Construct	Hypothetical Example
Predisposing Factors: the beliefs, knowledge, and attitudes of individuals that predispose them to certain aligned behaviors	Clinician knowledge of evidence in support of the CDSMP
Reinforcing Factors: the community norms, incentives, and infrastructures that shape and reinforce the predisposing factors	Frequency with which clinicians’ peers and administrators discuss the CDSMP
Enabling Factors: the immediate availability of individual and community resources required to carry out aligned behaviors	Availability of staff and technology to facilitate CDSMP referrals
Administrative Issues: the culture and priorities of a community or organization that determine, prescribe and facilitate changes to other factors	Organizational culture and mission that desires to keep people healthy
Policy Issues: the existing rules that require or prevent certain behaviors independent of motivations	Presence of organizational policies that limit external collaboration

### Measure development

As part of the parent project, we convened a regional stakeholder meeting in the Spring of 2015. Attendees (*n* = 52) were educated about the PRECEDE model before reviewing, critiquing, and piloting interview guides and surveys for both primary care clinicians and other stakeholders loosely based on the model. Because we hypothesized that the beliefs and attitudes of the two groups (clinicians vs other stakeholders) would be different and because we wanted to explore this, many questions and items were the same on both versions. Topically, the measures covered issues of interest to the community stakeholders and related to knowledge and beliefs about both the CDSMP specifically and community resources in general and other factors—such as resources, norms, and policies—expected to affect their implementation. To optimize response rates, the surveys were designed to be brief [32]. After synthesizing feedback, we posted revised versions of the guides and surveys for final stakeholder approval on a web-based project management tool (Basecamp). The Mayo Clinic Office of Community Engagement in Research approved the final versions of the interview guides and surveys and all study procedures. Copies of all measures are available in the Additional file 1: Online Supplement.

### Participants and recruitment

For the qualitative component, we elected to interview 30 participants. We chose this sample size because we were familiar with the context and relevant stakeholders through prior collaborative work and we were confident it would be both feasible and sufficient to capture the breadth of insights. ALL recruited the participants, which consisted of 15 primary care clinicians (“clinicians”) and 15 non-primary care clinician stakeholders (“stakeholders”). Clinicians were actively practicing physicians or nurse practitioners in the region. Stakeholders held a variety of titles (e.g. public health, patient, community agency, researcher, administrator) but were not actively practicing primary care; they were also known to have personal experience or special insight relevant to EBP implementation from either a community or healthcare perspective. (see Table 2) Participants in the quantitative component were also primary care clinicians practicing in the region (physicians, nurse practitioners, or physician assistants) and other stakeholders. Although most non-clinician stakeholders worked and resided within the region, some (e.g. state level stakeholders from the Department of Health) did not. Primary care clinicians for the quantitative analysis were recruited by contacting employing health systems (*n* = 5). A purposeful sample of other informed stakeholders was recruited through convenience sampling of currently engaged individuals (e.g. from the stakeholder meeting) and the contacts they recommended and provided. Six professional and 4 community researchers constituted the multidisciplinary research team. All community researchers were local professionals known to the PI from prior collaborative work. They had pre-existing expertise and experience in EBP implementation as part of their professional roles and were recruited personally by the PI after being given a description of the anticipated requirements. Prior to study initiation they received training in the protection of human subjects and the conduct of qualitative interviews. Throughout the study the community researchers reported back to a larger group of stakeholders affiliated with the parent project who were not directly involved in the research conduct (this was so the other stakeholders could proceed with the “action” component of the parent project).

**Table 2:** Description of interview participants

Primary care clinicians		Other Stakeholders (Constituency Represented)	
C101	Female, internal medicine physician who oversees nurse care coordinators for large system	S001	Endocrinologist and researcher with expertise in patient self-management (healthcare system structure informant)
C102	Male, family medicine physician who is on clinical practice committee for large system	S002	Health services researcher and patient familiar with CDSMP (healthcare system structure informant)
C103	Female, internal medicine physician at urban community practice of large system	S003	Coordinator of EBPs for Area Agency on Aging and CDSMP leader (funded to implement EBPs)
C104	Female, internal medicine physician at urban community practice of large system	S004	Executive Director of Area Agency on Aging (funded to implement EBPs)
C105	Male, internal medicine physician leader in population health of large system	S005	Health services researcher with expertise in chronic care delivery (healthcare system structure informant)
C106	Female, family medicine physician leader in population health for smaller system	S006	Director of community outreach for small rural hospital affiliated with large health system (healthcare system structure informant)
C107	Female, family medicine physician at urban community practice of large system	S007	Public health employee tasked with facilitating clinic-community linkages in rural county (funded to implement EBPs)
C108	Male, family medicine physician at urban community practice of large system	S008	Employee of state Department of Human Services with role in CDSMP implementation (funded to implement EBPs)
C109	Male, internal medicine physician at urban free clinic for underserved	S009	Nurse care coordinator at rural site in large health system (healthcare system structure informant)
C110	Female, family medicine nurse practitioner at rural clinic in small health system	S010	Director of community outreach for smaller health system and CDSMP leader (healthcare system structure informant)
C111	Male, family medicine physician assistant at rural clinic in small health system	S011	Director of community organization and CDSMP leader (funded to implement EBPs)
C112	Male, family medicine physician at rural clinic for large health system	S012	Nurse supervisor for nurse care coordinators in large health system (healthcare system structure informant)
C113	Female, family medicine physician and medical director of rural clinic for large system	S013	CDSMP leader at rural rehabilitation center (funded to implement EBPs)
C114	Male, family medicine physician at rural community practice of large system	S014	Employee of state Department of Health with role in EBP implementation and CDSMP leader (funded to implement EBPs)
C115	Male, family medicine physician at urban community clinic	S015	Community leader working with healthcare and focused on health in urban development (healthcare system structure informant)

### Procedure

Over a three-month period, an experienced qualitative researcher (JE) conducted semi-structured face-to-face and phone interviews of the 15 non-clinician stakeholders. During this same time, 4 community researchers conducted similar interviews of the clinicians (each conducting 2–4 interviews). This approach was taken because we anticipated the clinician interviews to be less variable, more interesting, and less challenging technically for the community researchers. Interviews took approximately 30 min each; they were audiotaped and transcribed for analysis. Surveys of clinicians were distributed electronically or printed and distributed in person or by postal mail via complete or convenience sampling, depending on the preference of the participating healthcare systems. When possible, we sent printed surveys by postal mail to non-responding clinicians who received electronic surveys. Other stakeholders received their surveys electronically via email; we sent electronic reminders to non-responders. We entered and stored all responses in a secure (RedCAP) [33] database for analysis.

### Analysis

We conducted analyses in two phases (qual before quan) so as not to bias the qualitative interpretations. Using the PRECEDE framework, two team members (SD and JE) working independently and in duplicate, coded all the interviews. Specifically, interviewee comments were categorized into a priori codes of Predisposing, Enabling, and Reinforcing Factors, and Administrative and Policy Issues. After the initial coding, the team collectively reviewed transcripts to reconcile individual coding interpretations and confirm theoretical saturation. Narrative summaries were then distilled into brief and representative summary phrases and imported into one of two 5 (codes) by 15 (participants) frameworks for both clinicians and other stakeholders as appropriate. These frameworks were developed to provide a visual and actionable overview of the emerging information that the implementing stakeholders could understand and apply in the action phase of the parent project. To obtain a richer understanding of the key issues, a team of three experienced qualitative researchers (ALL, JE, KS) reviewed the interview content and explored the ways in which the various PRECEDE factors interacted within and across participant groups. This process was used to develop additional, emergent themes and to gain more generalizable insights.

During the quantitative phase of the analysis, we categorized survey items—before looking at the data—by whether they were likely to generate insight that could triangulate with the qualitative PRECEDE codes. We calculated response rates as applicable and used standard descriptive statistics to characterize the clinician and non-clinician stakeholders and their responses. We tested for differences in proportions between groups with chi square tests of association. We followed a similar procedure for the quantitative assessment of the emergent themes with the exception that—for those themes that did not clearly correspond to a priori survey items—we constructed variables and analyses as feasible and appropriate (as determined by the authors). We conducted all quantitative analyses with SAS statistical software. Finally, we compared the collective insight from both the qualitative and quantitative analyses, judged for conceptual consistency, and took note of the ways in which methods and data sources confirmed, explained, and/or contradicted one another. Figure 1 shows an overview of the process we used to collect, analyze, and make sense of the data.

## Results

### Qualitative component

#### Demographics

We interviewed primary care clinicians from the three largest healthcare provider systems in Southeast Minnesota (a clinician from the fourth system declined to participate and we were unable to contact a clinician from the fifth system). Most were physicians that had some understanding of what the CDSMP was and the evidence supporting it. Only one, however, had successfully referred a patient to the program. The other interviewees were a diverse group of local and state level stakeholders. Eight were from the community and seven worked within a healthcare provider system in either a research or clinical capacity (Table 2).

#### PRECEDE analysis

**Fig. 2:**
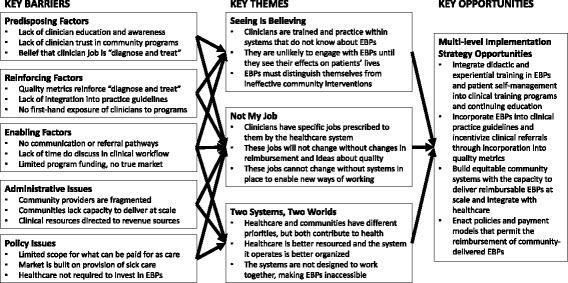
Summary of PRECEDE barriers identified, emergent themes, and implementation strategies of potential value

Overall, we found relevant discussion mapping to all PRECEDE codes in both participant groups and examples of key barriers identified, organized by PRECEDE construct, are presented in Fig. 2. For a detailed table of representative quotes from clinician and stakeholder interview participants organized by PRECEDE construct, see the Additional file 1: Online Supplement. Here, we emphasize the overarching and emergent themes that arose from the deep exploration of the within and cross group interactions of these codes: namely, “*Two Systems, Two Worlds*,” “*Not My Job*,” and “*Seeing Is Believing*.”

#### Emergent themes analysis

The theme of *Two Systems, Two Worlds* highlighted the importance of key policy and administrative issues in determining the different priorities of healthcare and the community. Superficially, it was supported by the fact that non-clinician stakeholders within the healthcare system seemed to offer insights more similar to those of clinicians than to those of their community-based counterparts in regards to both tone and topic. This discordance appeared to be rooted in key policy and administrative issues that determined that the priorities of—and associated resources and types of knowledge available to—participants within and outside of the healthcare system be different. Key community stakeholders, for example, were familiar with the CDSMP and other EBPs because of Federal and state-level policies and initiatives that funded and/or encouraged their particular implementation of them. This was especially true at the local Area Agency on Aging, where EBP implementation was the entire focus of one employee’s job. She even described the policy-enacted support she had at her disposal to do this work, claiming it was too directive and limited:

*“So a portion of the [EBP] workshops that go on are funded under Older Americans Act Title IIID funding, which is, that funding has to be used specifically for evidence-based health promotion programs but they also have to be geared toward seniors. So that’s a limitation of that. And then another portion of workshops going on, the [providing] organizations are just taking it on as part of what they do as an organization.”*

This approach of engaging organizations to take programs on as “what they do” was a key strategy for scaling EBP implementation in the setting of fixed resources. And, as it turned out, the most accessible and willing partners were typically community-based non-profits. The activities these organizations undertook together to coordinate the training of leaders and licensing and delivery of programs seemed to contribute to the development of a “community system” of trust, familiarity, and purpose, and to the development of a common way of working. Perhaps consequently—and reflective of and reinforced by past efforts—the community stakeholders viewed the CDSMP as something they mostly did (or were trying to do) independent of the healthcare system. Ironically, this was not their desire, and many interviewees highlighted the need for clinical referrals to the programs for success and sustainability. Indeed, clinical integration had apparently always been the hope and expectation of EBP implementation; but, many were giving up on that ambition. As one reflected:

*“Nationally, when all of these evidence-based programs were funded by the Older Americans Act, the thought was that we were gonna educate doctors and they were gonna be telling patients about these programs and classes, but that’s not realistic. You know their time, ah, is so prescribed in many of the clinics across the country that that’s just not a feasible approach.”*

The clinicians we interviewed uniformly agreed that their time was heavily prescribed, and that making referrals to the CDSMP and other EBPs was not part of what they were being encouraged to do. In that respect, it seemed that the policy and administrative issues of the healthcare system were not well matched to those of the community. As one clinician said:

*“Part of the issue [preventing clinicians from making referrals] may be you know there, there are so many things coming at, at us in healthcare these days. Including expectations and requirements about different ways to care for patients. About things we have to meet for metrics and measures.”*

Although they were often a source of burden and frustration, clinicians felt most responsible for these “metrics and measures”—typically based on clinical practice guidelines for disease screening and treatment protocols. The reason these measures were prioritized (while EBP referrals were not, for example) was that they were often tied to quality-based payment contracts. These complicated relationships formed the major priorities of the healthcare system and, thus, they helped to shape the jobs of clinicians.

Related to this, *Not My Job* emerged as a distinct theme to represent primary care clinicians’ apparent lack of interest in carrying out the acts of referring patients to the CDSMP and/or other community resources. Indeed, primary care clinicians often reported that nurses and care coordinators had more time and more expertise when it came to discussing these sorts of opportunities with patients. As one physician said, “I am [only] trained in diagnosis and treatment.” Because, in the minds of most clinicians, the CDSMP was not thought of as diagnosis or treatment, it could have been hard for clinicians to conceptualize the program as part of what they do. Certainly, clinicians had no frame of reference for what actually happened in the CDSMP because it was not part of their education or training. Consequently, clinicians felt both a lack of responsibility for and capability in making referrals. As one physician said.

*“And the truth is that there are times I tell you that, you know I’m, I’m missing even I’m not gonna say more critical things but—but things I measure on. So…(sigh) is there, is there an automatic way to do this?”*

The idea of “automating” the referral process was often explicitly suggested as a way to save time. Implicitly, however, it seemed that clinicians proposed the idea as a polite way of deferring responsibility for and attention to the task. It also may have represented a lack of interest in integrating the program into treatment plans. Along these lines, clinicians were quick to request that any information about the community programs or feedback related to when a patient participated be brief. It was not clear, for example, that clinicians had considered or desired a scenario by which they would adapt their care plans based on the goals identified or the lifestyle or self-care changes made by patients through program participation.

A notable exception to this was clinician C113. As the medical director of a rural practice, she had had the opportunity to attend a meeting in which a CDSMP participant had talked about her experience in the program. The clinician then familiarized herself with the program’s curriculum and began referring some of her patients to the program. She had come to find so much value in the program, she was considering attending herself:

*It would be kind of fun, actually, just to wouldn’t it cuz I read the book, actually but just to sit in the class because, you know, I don’t have half these issues, but there still are things in they’re just lessons in life to learn, like, how to be happier and, you know, other things. So like I said, I have two [patients] that have gone through it and both of them have said that this is this has been a great program. So, again, I’m all in and if there’s other ways, like I said, that you think that we can get this information out, I’m, I’m more than willing to, you know, try to do that.*

In many ways, the enthusiasm and certainty with which C113 described the program’s value was similar to that of many community stakeholders. What all these individuals had in common—and what distinguished them from most clinicians—was first-hand experience with the program itself and with its participants. The importance of this as a factor in increasing trust in and confidence about the program’s value was consistent across clinician and stakeholder groups inside and outside the healthcare system. Indeed, it was those closest to the program that were most able to articulate how it helped people, often citing powerful anecdotes of participant success. As one program leader (S010) said:

*The one thing that sticks out is people [with chronic conditions] think they’re alone out there. So a lot of people with tears in their eyes would say, “I thought I was the only one feeling like this.” And they realize that they’re not. That everybody feels all of these things…no matter if it’s diabetes, depression whatever it might be, they’re all feeling the same thing. And the dynamics of the group interaction is, is the meeting. We’re there as leaders but we’re really just a guide. And they pretty much help each other. And they interact they hold each other accountable. We had one gentleman and wife who came. They came for him; he wasn’t doing his homework. The other people in the class kept him accountable. And said, “Hey, you need to do this; your wife is emotional about this.” And his wife was really the one who benefited the most from it as a care taker. And so when she left the last session she came up to me and she was crying. And she said, “Thank you for saving our marriage.” So it so it can have really profound effects.*

These effects were difficult for clinicians to fully appreciate, even after familiarizing themselves with evidence summaries of the program’s value. “*Seeing is Believing*,” captured this idea and represented the fact that clinicians who had no first-hand exposure to the CDSMP were forced to develop an impression through use of their imaginations. These impressions seemed to be reinforced by experiences with or impressions of other programs or interventions that patients pursued outside of the healthcare system. This connection raised concern in the minds of some that clinicians—especially “old fogeys”—would think of the CDSMP as “wishy washy” or alternative to effective care. Indeed, one clinician perceived the program as something that would be best suited for patients that were noncompliant with clinical care:

*“So for instance somebody who doesn’t necessarily trust medications or even though they may really need medications based on my recommendations wanna avoid meds, avoid drugs, find a more holistic approach to it. And I find that my knowledge there is fairly limited. So the yoga, the tai chi, and what not…”.*

In some ways, it seemed that the healthcare system was well positioned to reinforce a skepticism among clinicians about the value of all things outside its walls. Although this could be perceived as a reasonable and protective heuristic in many cases, it might have also predisposed clinicians to distrust EBPs and, potentially, focus less on the work that patients had do to self-manage their health. A key priority of the quantitative component was to further explore the validity of these ideas.

### Quantitative component

#### Demographics

In regards to that effort, we were successful in surveying 190 primary care clinicians from 4 of the 5 healthcare provider systems (and 9 of the 11 counties) in Southeast Minnesota. We did not receive responses to requests to contact clinicians from the smallest system, although there were few clinicians there, representing less than 5% of clinicians in the region. Approximately half (52%) of the clinicians practiced in an urban/suburban community, while the rest were located in more rural settings. The response rate for the largest of the health systems was 37% (174/474). Response rates for the other systems were not calculable but were believed to range from 5 to 50% (the surveys were handed out in person to attendees of institutional healthcare provider meetings and exact denominators are not known). Through convenience and snowball sampling procedures, we distributed surveys to 167 non-clinician stakeholders. Of these, 88 (53%) completed the survey, with respondents representing every county in SE MN. Table 3 describes the survey participants.

**Table 3:** Characteristics of survey study participants

Participant Characteristic	N (%)
Primary Care Clinicians *N* = 190	
Type of Practice	
Family Practice	101 (53.2%)
Internal Medicine	50 (26.3%)
Other (urgent care, ED)	27 (14.2%)
Degree	
MD/DO	107 (56.3%)
PA	11 (5.8%)
NP	49 (25.8%)
Other	9 (4.7%)
Other Stakeholders *N* = 88	
Role/interest in improving health in SEMN	
Health care employee that may refer patients to community resources	19 (22%)
Health care employee not likely to refer patients to community resources	12 (14%)
Public health	14 (16%)
Community-based agency or non-profit	16 (18%)
Patient	8 (9%)
Researcher	7 (8%)
Payer/Insurer	1 (1%)
Funder/Philanthropist	1 (1%)
Contractor	1 (1%)
Volunteer	2 (2%)
Other	7 (8%)

^*^Missing responses not counted in percentages

#### PRECEDE analysis

We selected 8 survey items to represent four of the pre-determined PRECEDE constructs (we were not confident that any of the items corresponded to relevant policy issues). Broadly, the questions asked both clinicians and stakeholders about their perceptions of barriers to clinical referrals to the CDSMP and other community resources (see Table 4). Overall, respondents (260, 96%) overwhelmingly agreed that community resources were integral to effective primary care. They also highlighted lack of clinician education and awareness about the CDSMP (203, 73%) and practical challenges in referring to community resources (220, 79%) as key barriers. Importantly, both clinicians and stakeholders (63 and 74%, respectively) felt that community resources were underutilized and underemphasized in their organizations. However, clinicians were twice as likely to emphasize the need for community resources to be trustworthy and reliable than their stakeholder counterparts (67% vs 34%, respectively; p = < 0.001). Additional details about the PRECEDE survey results, organized by construct and participant group are presented in Table 4.

**Table 4:** Dichotomized responses to PRECEDE survey items

Survey Measure	Total (*n* = 278)	Clinicians (*n* = 190)	Stakeholders (*n* = 88)	*P* value
Predisposing Factors				
Believe community resources are important parts of effective primary care^a^	260 (95.9%)	179 (95.2%)	81 (97.6%)	0.36
Believe community resources need to be reliable and trustworthy^b^	153 (56.7%)	125 (66.8%)	28 (33.7%)	< 0.01
Believe lack of education and awareness about program is barrier to CDSMP referral^c^	203 (73.0%)	134 (73.2%)	69 (86.3%)	0.02
Reinforcing Factors				
Believe community resources, if suggested, are not likely to be used by patients^a^	43 (15.9%)	33 (17.6%)	10 (12.0%)	0.25
Believe community resources are not accessible^b^	193 (71.5%)	111 (59.4%)	82 (98.8%)	< 0.01
Enabling Factors				
Believe easy to make referrals to community resources if desired^a^	43 (15.9%)	32 (17.0%)	11 (13.3%)	0.43
Administrative Issues				
Believe community resources are emphasized and encouraged in my setting^d^	114 (42.2%)	76 (40.4%)	38 (46.3%)	0.37
Believe community resources are underutilized in my setting^d^	180 (66.7%)	119 (63.3%)	61 (74.4%)	0.08

^a^Missing 7 responses (2 clinicians); ^b^Missing 8 responses (3 clinicians); ^c^Missing 15 responses (7 clinicians); ^d^Missing 8 responses (2 clinicians)

#### Emergent themes analysis

To explore the validity and nuance of the theme of *Two Systems Two Worlds*, we selected out the stakeholders who worked within the healthcare system and combined them with the clinicians to create a “healthcare system group” of 223 participants. We then used the cross-group comparisons for all PRECEDE factors between this group and the community system group of 55 participants (e.g. the stakeholders that did not work within the healthcare system). This analysis did not change impressions greatly, but did reinforce key differences between community and healthcare-based stakeholders. Specifically, healthcare system stakeholders were significantly less aware (*p* < 0.001), less trusting (*p* = 0.02), and less concerned about the accessibility of (p < 0.001) community resources than their community-based counterparts.

We identified 3 survey measures that corresponded to the theme of *Not My Job* and analyzed these in the same way as the PRECEDE factors. Specifically, the items explored the extent to which clinicians desired or were willing to take on the work required for clinical integration of the CDSMP. Consistent with the theme, only a minority of clinicians (62, 34%) expressed concern about the lack of a system for making referrals and few (26, 14%) expressed a clear desire to receive feedback if and when their patients participated in the program. Not surprisingly, the vast majority (151, 84%) of clinicians reported that any system pursued to initiate referrals must fit into the electronic workflow. Non-clinician stakeholders expressed significantly less extreme perceptions of the *Not My Job* measures in all 3 cases.

Finally, we used the clinician distributions of all survey items representing the Predisposing factors as well as an item assessing clinicians’ perceptions of whether community resources were underutilized in their practice across levels of exposure (some vs none) to the CDSMP to represent *Seeing is Believing*. We found that clinicians with exposure to the CDSMP were significantly less likely to cite education and awareness as a barrier to referral (*p* < 0.0001). Exposure to the CDSMP was not significantly associated with better perceptions of community resources in general, however. This supports the idea that the CDSMP and other EBPs may not be viewed in the same light as other, more typical and “trustworthy” community resources. Other details about the quantitative analyses of the emergent themes are presented in Table 5.

**Table 5:** Emergent themes analyses

Two Systems Two Worlds				
Survey Item	Total (n = 278)	Healthcare system (*n* = 223)	Community system (*n* = 55)	*P* value
Predisposing Factors				
Believe community resources are important parts of effective primary care^a^	260 (93.5%)	209 (93.7%)	51 (92.7%)	0.39
Believe community resources need to be reliable and trustworthy^b^	153 (55.0%)	131 (58.7%)	22 (40.0%)	0.02
Believe community resources are something I am aware of and educated about^b^	78 (28.1%)	74 (33.2%)	4 (7.3%)	< 0.01
Reinforcing Factors				
Believe community resources, if suggested, are not likely to be used by patients^a^	43 (15.5%)	39 (17.5%)	4 (7.3%)	0.07
Believe community resources are not accessible^b^	193 (69.4%)	142 (63.7%)	51 (92.7%)	< 0.01
Enabling Factors				
Believe easy to make referrals to community resources if desired^a^	43 (15.5%)	36 (16.1%)	7 (12.7%)	0.60
Administrative Issues				
Believe community resources are emphasized and encouraged in my setting^c^	114 (41.0%)	89 (39.9%)	25 (45.5%)	0.28
Believe community resources are underutilized in my setting^c^	180 (64.7%)	146 (65.5%)	34 (61.8%)	1.00
Not My Job				
Survey Item	Total (n = 278)	Clinicians (n = 190)	Stakeholders (n = 88)	*P* Value
Believe lack of feedback about patient participation is barrier to referral^d^	62 (22.3%)	26 (14.2%)	36 (45.0%)	< 0.01
Believe lack of an electronic referral system is barrier to referral^d^	105 (37.8%)	62 (33.9%)	43 (53.8%)	< 0.01
Believe referral process, if pursued, must be integrated into work flow^e^	209 (75.2%)	151 (84.4%)	58 (69.9%)	< 0.01
Seeing is Believing				
Survey Item	All clinicians (*n* = 186^f^)	Clinicians with CDSMP exposure (*n* = 38)	Clinicians w/out CDSMP exposure (*n* = 148)	*P* value
Believe community resources are important part of primary care	177 (95.2%)	35 (92.1%)	142 (96.0%)	0.33
Believe community resources need to be reliable and trustworthy^g^	123 (66.5%)	23 (62.2%)	100 (67.6%)	0.53
Believe education and awareness about CDSMP is barrier to referral^h^	134 (73.2%)	7 (18.9%)	127 (87.0%)	<.0001
Believe community resources are underutilized in their setting	118 (63.4%)	23 (60.5%)	95 (64.2%)	0.68

^a^Missing 7 responses, 4 from healthcare; ^b^Missing 8 responses, 5 from healthcare; ^c^Missing 8 responses, 4 from healthcare; ^d^Missing 15 responses, 7 from clinicians; ^e^Missing 16 responses, 11 from clinicians; ^f^4 clinicians did not respond to question to ascertain exposure; ^g^Missing 1 response for a clinician with exposure to CDSMP; ^h^Missing 3 responses (1 from a clinician with exposure to CDSMP)

## Discussion

### Our findings

We sought to understand, categorize, and richly describe key challenges and opportunities related to integrating EBPs into routine primary care practice in the United States as part of a parent, participatory action research project. Three themes emerged from this investigation that help explain why primary care and community-based programs exist in disconnected worlds.

### Limitations and strengths

Our study has several weaknesses and, partly for this reason, it should be viewed as exploratory. Small numbers of interview participants among some stakeholder constituencies, although representative of Southeast Minnesota, could have prevented us from reaching a generalizable saturation of insights. Sampling bias, due in part to sampling procedures and low response rates, may threaten the validity of the survey data. Additionally, our surveys were developed to explore topics consistent with PRECEDE constructs and to triangulate with qualitative data. They were not designed to measure PRECEDE constructs themselves, nor was any reliability or validity testing done. Although not feasible for our study, the survey likely would have been more useful had it been designed after completion of the qualitative component. Our focus on a large, 11-county region of Southeast Minnesota, albeit relevant to our purpose, may also threaten the applicability of our findings. The validity of our findings is strengthened, however, by the sampling of an entire geographic region, our conduct of quantitative analyses after and in confirmation of qualitative data, and the congruence of findings across methods and participants. Our study also adds to the body of literature related to EBPs in that—while many studies focused on EBP effectiveness, acceptability, and value, have been conducted [34–37]—to our knowledge, this is the first empirical, theory-guided exploration of the feasibility of clinical integration of community-based EBPs. In that respect, this study provides timely evidence to stakeholders seeking to scale EBPs and improve population health.

## Conclusions

Our findings suggest that key policy and administrative issues have played important roles in prescribing—in a very fragmented and siloed way—the work that healthcare and the community are “supposed to do” to contribute to health. Two separate systems with distinct cultural identities coexist and do not understand each other well. Our work supports prior laments describing healthcare as a “sick care” system [38], for example, but adds deeper understanding of the pathways by which healthcare is directed to focus somewhat exclusively on the activities of “diagnosis and treatment.” It also begins to articulate the role EBPs may play in person-centered and evidence-based efforts to improve population health. Indeed, to the extent “well-care” is needed and can be operationalized through the delivery of EBPs, the community appears capable and motivated to contribute. Its capacity to do this at scale and in a way healthcare will understand and accept—and eventually embrace, coordinate with, and seamlessly integrate—is likely to require bidirectional education and engagement, as well as policy and system-level changes (see Fig. 2).

### Practice implications

Stakeholders guiding efforts to transform healthcare delivery in the United States should consider our findings. Specifically, the Department of Health and Human Services has made CDSMP implementation a key goal in its framework for improving the health of patients with multiple chronic conditions [39], and the CDC and the Centers for Medicare and Medicaid Services (CMS) have rolled out initiatives to implement and pay for the Diabetes Prevention Program, respectively. For the most part, these and other approaches to EBP implementation have relied on community-based and publicly-funded dissemination channels, and only assumed the participation of primary care. Our findings suggest that this strategy is unlikely to penetrate the healthcare system or influence its priorities. Indeed, to the extent disease experts—who may be trained only in the activities of “diagnosis and treatment”—continue to develop and inform the “metrics and measures” that determine what primary care clinicians know, do, and get paid for [40, 41], actual referrals to EBPs and incorporation of them into treatment plans are unlikely to come at scale.

### Future directions

For this and other reasons, some might argue that the potential benefits of integrating EBPs into clinical care are not worth the effort and resources. We are wary of this logic and warn of the potentially negative consequences of community-based implementation that is entirely independent of healthcare. For example, we uncovered numerous examples of clinician distrust of EBPs and concerns about the extent to which these programs are perceived to “compete with” clinical care. If explicit efforts are not undertaken to engage clinicians—potentially through experiential learning and detailing—we foresee a scenario by which clinicians become less supportive of and potentially opposed to EBPs. This would undermine the community’s efforts. We believe future work should focus on the development and testing of multi-level implementation strategies that seek to engage clinicians and broaden the priorities of healthcare. Such strategies should be aligned to counteract the barriers identified here (see Fig. 2) [42]. They should promote and reward the establishment of clinic-community partnerships and align with system and reimbursement model transformations presently occurring in the United States [43]. Specifically, the value of reorganizing, reframing, and repurposing the community and articulating its potential and non-duplicative role as a “well-care” system of EBPs should be explored. We believe this could facilitate the creation and orient the function of scalable clinic-community partnerships that improve population health. Indeed, this is the approach our own community-partnered team has taken as part of the parent project. Thus far, it has spurred the creation of a “hub-based” [44] model for EBP implementation and an accompanying technology (www.wellconnectsemn.org). We are engaging with learning collaboratives convened by the National Council on Aging and others interested in similar approaches to explore opportunities for refinement and evaluation.

## Additional files

Additional file 1: a) Primary Care Clinician Survey. b) Stakeholder Survey. c) Participant Interview Guide. d) Sample Participant Quotes by PRECEDE Construct (PDF 784 kb)

## Abbreviations

AHRQ: The Agency for Healthcare Research and Quality
CDC: The U.S. Centers for Disease Control and Prevention
CDSMP: The Chronic Disease Self-Management Program
CMS: The U.S. Centers for Medicare and Medicaid Services
DHHS: The U.S. Department of Health and Human Services
EBPs: Evidence-based health promotion programs
PRECEDE: Predisposing, Reinforcing, and Enabling Constructs in Diagnosis and Evaluation and the associated implementation planning model
