# Supplementary material for: Integrating community-based health promotion programs and primary care: a mixed methods analysis of feasibility
Source: BMC Health Serv Res. 2018 Jan 31;18:72. doi: 10.1186/s12913-018-2866-7 (PMC5793407; doi:10.1186/s12913-018-2866-7)
Supplement: Supplementary file 1 — a) Primary Care Clinician Survey. b) Stakeholder Survey. c) Participant Interview Guide. d) Sample Participant Quotes by PRECEDE Construct (PDF 784 kb) [file 12913_2018_2866_MOESM1_ESM.pdf]

SUPPLEMENTARY INFORMATION

Integrating Community-based Health Promotion Programs into Primary Care: a mixed methods analysis of feasibility

Contents:

Primary Care Clinician Survey

Stakeholder Survey

Participant Interview Guide

Sample Participant Quotes by PRECEDE Construct

Barriers to Community Resource Utilization: Primary Care Clinician Survey

The CDSMP Implementation Project

The Southeast Minnesota Partnership for Community-Based Health Promotion is a regional collaborative of stakeholders from public health, health care, and community partners. It is funded by the Catalyst Initiative of the George Family Foundation and by the Mayo Clinic Office of Community Engagement in Research.

Our aim is to establish a robust infrastructure for linking clinical care to evidence-based community resources for health promotion and chronic disease management. We are also researching this process so that we will know more about the ways in which diverse stakeholders partner together to improve population health.

As this is a research study, your participation is voluntary. Completing this survey indicates your agreement to participate in this research study. To respect your privacy, we will remove any information that may allow us to connect you to your responses upon receiving your completed questionnaire. If you have any questions or comments about this survey or study, please call: 1-[illegible]-XXX-XXXX

We desire to create a sustainable system that meets the needs and preferences of primary care clinicians across Southeast Minnesota. Your thoughts and suggestions are very important to us.

Thanks for answering these questions!

Some Questions About Community Resources:

Community resources include any services, programs, or activities offered outside of the health care system that can benefit the health and wellness of patients. Please state the extent to which you disagree or agree with the following statements. [please check ☑ one response for each statement]

1. In general, I believe that community resources…	Strongly Disagree	Disagree	Slightly Disagree	Slightly Agree	Agree	Strongly Agree
a. …are important components of effective primary care	☐	☐	☐	☐	☐	☐
b. …need to be reliable and trustworthy	☐	☐	☐	☐	☐	☐
c. …are not accessible for my patients	☐	☐	☐	☐	☐	☐
d. …are underutilized in my practice	☐	☐	☐	☐	☐	☐
e. …make practice less evidence-based	☐	☐	☐	☐	☐	☐
f. …are not things I am aware of or educated about	☐	☐	☐	☐	☐	☐
g. …are easy for me to make referrals to when desired	☐	☐	☐	☐	☐	☐
h. …are emphasized and encouraged in my practice environment	☐	☐	☐	☐	☐	☐

<u>Some Questions About the Chronic Disease Self-Management Program:</u>

The Chronic Disease Self-Management Program (CDSMP) (also known as "Living Well with Chronic Conditions") is an evidence-based and manual-guided program led by trained peer leaders. It improves outcomes and reduces costs and is promoted by the Centers for Disease Control, the Agency for Healthcare Research and Quality, and the Minnesota Department of Health, among others. Participants learn flexible self-management strategies for dealing with chronic physical and mental health conditions over 6 weekly sessions.

Please answer the following questions <u>about the Chronic Disease Self-Management Program</u>.

7. What is your level of exposure to the CDSMP (also known as "Living Well with Chronic Conditions")? [please check ☑ one]

☐$_1$ I have never heard of this program
☐$_2$ I have heard of the program but am not aware of its availability in my area
☐$_3$ I have heard of the program but have not recommended it to any of my patients
☐$_4$ I have recommended it to one or a few of my patients
☐$_5$ I routinely refer patients to this program

8. What of the following are barriers to you referring a patient to the CDSMP? [please check ☑ one or more]

☐$_1$ Lack of awareness or education
☐$_2$ Lack of program availability in my area
☐$_3$ Lack of follow-up or feedback about program completion or effectiveness
☐$_4$ Lack of a simple and reliable system for making referrals
☐$_5$ Lack of belief in the program's value
☐$_6$ Lack of clarity on which patients would most benefit
☐$_7$ Other (specify)____

Some questions about the Community Resource Referral Infrastructure

We aim to build a health system-agnostic infrastructure for linking clinical care to evidence-based community resources. Please state the extent to which you disagree or agree with the following statements about what this infrastructure should accomplish. [please ☑ one response for each statement].

9. In general, I believe the community resource referral infrastructure…	Strongly Disagree ↓	Disagree ↓	Slightly Disagree ↓	Slightly Agree ↓	Agree ↓	Strongly Agree ↓
a. …should only include resources that are evidence-based	☐	☐	☐	☐	☐	☐
b. …should be centralized for the region and searchable by location	☐	☐	☐	☐	☐	☐
c. …should be automated into the electronic orders workflow	☐	☐	☐	☐	☐	☐
d. …should provide information about what the resources are and who they will benefit	☐	☐	☐	☐	☐	☐
e. …should return key information about resource use and completion for documentation	☐	☐	☐	☐	☐	☐
f. …should ensure resources are not biased toward any particular health system	☐	☐	☐	☐	☐	☐

10. We want to create a community resource referral infrastructure that meets your needs. If you have one key suggestion for how we can do this, please state it here:

____

____

Some Questions About You:

1. Which type of primary care do you practice in Southeast Minnesota?

☐1 Family Practice
☐2 Internal Medicine
☐3 Other (specify) ______________________________
☐4 I do not practice primary care in Southeast Minnesota

2. What is your degree within your healthcare profession? [please check ☑ one]

☐1 MD/DO
☐2 PA
☐3 NP
☐4 Other (specify) ______________________________

3. With which health care system are you primarily affiliated? [please check ☑ one]

☐1 Allina
☐2 Mayo Clinic Health System
☐3 Olmsted Medical Center
☐4 Gundersen Lutheran
☐5 Veteran Affairs
☐6 Winona Health
☐7 Other/Private (specify) ______________________________

4. In which Southeast Minnesota County do you primarily practice? [please check ☑ one]

☐1 Rice
☐2 Goodhue
☐3 Wabasha
☐4 Steele
☐5 Dodge
☐6 Olmsted
☐7 Winona
☐8 Freeborn
☐9 Mower
☐10 Fillmore
☐11 Houston

Some Questions About Your Practice:

1. Which best describes your practice setting?

☐$_1$ Community with population <5,000
☐$_2$ Community with population 5,000-20,000
☐$_3$ Community with population 20-000=50,000
☐$_4$ Community with population >50,000

2. Please indicate if your practice particularly serves any of the following patient populations? [please check ☑ one or more]

☐$_1$ Rural, underserved
☐$_2$ Urban, underserved
☐$_3$ Low socioeconomic status
☐$_4$ Racial or ethnic minorities (specify)___________________________________
☐$_5$ Older adults
☐$_6$ None of the above particularly
☐$_7$ Unsure

3. We are looking for clinical champions to help develop a system for integrating evidence-based community resources into routine primary care in Southeast Minnesota. Would you be interested in learning more or partnering with us in this effort? [please check ☑ one]

☐$_1$ No, not at this time
☐$_2$ Yes, my contact information is: (specify name and contact)

____

Thank you for completing this survey!

Barriers to Community Resource Utilization: Stakeholder Survey

The CDSMP Implementation Project

The Southeast Minnesota Partnership for Community-Based Health Promotion is a regional collaborative of stakeholders from public health, health care, and community partners. It is funded by the Catalyst Initiative of the George Family Foundation and by the Mayo Clinic Office of Community Engagement in Research.

Our aim is to establish a robust infrastructure for linking clinical care to evidence-based community resources for health promotion and chronic disease management. We are also researching this process so that we will know more about the ways in which diverse stakeholders partner together to improve population health.

As this is a research study, your participation is voluntary. Completing this survey indicates your agreement to participate in this research study. To respect your privacy, we will remove any information that may allow us to connect you to your responses upon receiving your completed questionnaire. If you have any questions or comments about this survey or study, please call: 1-507-XXX-XXXX

We desire to create a sustainable system that meets the needs and preferences of all stakeholders in Southeast Minnesota. Your thoughts and suggestions are very important to us.

Thanks for answering these questions!

©The SE MN Partnership for Community-Based Health Promotion

Some Questions About Community Resources:

Community resources include any services, programs, or activities offered outside of the health care system that can benefit the health and wellness of patients. Please state the extent to which you disagree or agree with the following statements. [please check ☑ one response for each statement]

1. In general, I believe that community resources…	Strongly Disagree	Disagree	Slightly Disagree	Slightly Agree	Agree	Strongly Agree
a. …are important components of effective primary care	☐	☐	☐	☐	☐	☐
b. …need to be reliable and trustworthy	☐	☐	☐	☐	☐	☐
c. …are not accessible to most people that need them	☐	☐	☐	☐	☐	☐
d. …are underutilized by clinicians in my community	☐	☐	☐	☐	☐	☐
e. …are not based in scientific evidence	☐	☐	☐	☐	☐	☐
f. …are not things I am aware of or educated about	☐	☐	☐	☐	☐	☐
g. …are structured to easily receive clinician referrals	☐	☐	☐	☐	☐	☐
h. …are emphasized and encouraged in my community	☐	☐	☐	☐	☐	☐

Some Questions About the Chronic Disease Self-Management Program:

The Chronic Disease Self-Management Program (CDSMP) (also known as "Living Well with Chronic Conditions") is an evidence-based and manual-guided program led by trained peer leaders. It improves outcomes and reduces costs and is promoted by the Centers for Disease Control, the Agency for Healthcare Research and Quality, and the Minnesota Department of Health, among others. Participants learn flexible self-management strategies for dealing with chronic physical and mental health conditions over 6 weekly sessions.

Please answer the following questions <u>about the Chronic Disease Self-Management Program</u>.

7. What is your level of exposure to the CDSMP (also known as "Living Well with Chronic Conditions")? [please check ☑ one]

☐1 I have never heard of this program
☐2 I have heard of the program but am not aware of its availability in my area
☐3 I have heard of the program but have not recommended it to anyone
☐4 I have recommended it to one or a few people that I know
☐5 I routinely recommend this program to people I know

8. What of the following do you think are barriers to clinicians referring patients to the CDSMP? [please check ☑ one or more]

☐1 Lack of clinician awareness or education
☐2 Lack of program availability in the clinician's community
☐3 Lack of follow-up or feedback to the clinician when referrals are made
☐4 Lack of a simple and reliable system for making referrals to the program
☐5 Lack of clinician belief in the program's value
☐6 Lack of clarity to clinicians on which patients would should be referred
☐7 Other (specify)____

Some questions about the Community Resource Referral Infrastructure

We aim to build a health system-agnostic infrastructure for linking clinical care to evidence-based community resources. Please state the extent to which you disagree or agree with the following statements about what this infrastructure should accomplish. [please ☑ one response for each statement].

9. In general, I believe the community resource referral infrastructure…	Strongly Disagree	Disagree	Slightly Disagree	Slightly Agree	Agree	Strongly Agree
a. …should only include resources that are evidence-based	☐	☐	☐	☐	☐	☐
b. …should be centralized for the region and searchable by location	☐	☐	☐	☐	☐	☐
c. …should be automated into the electronic orders workflow	☐	☐	☐	☐	☐	☐
d. …should provide information about what the resources are and who they will benefit	☐	☐	☐	☐	☐	☐
e. …should return key information about resource use and completion for documentation	☐	☐	☐	☐	☐	☐
f. …should ensure resources are not biased toward any particular health system	☐	☐	☐	☐	☐	☐

10. We want to create a community resource referral infrastructure that meets your needs. If you have one key suggestion for how we can do this, please state it here:

____

____

Some Questions About You:

1. Which best describes your stakeholder role in Southeast Minnesota? [please check ☑ one]

   ☐1 Health Care System or Provider
   ☐2 Public Health
   ☐3 Community-based Agency or Non-Profit
   ☐4 Patient
   ☐5 Researcher
   ☐6 Policy-maker
   ☐7 Payer/Insurer
   ☐8 Employer
   ☐9 Funder/Philanthropist
   ☐10 Contractor
   ☐11 Volunteer
   ☐12 Media
   ☐13 Other (specify)

2. What is your title or job description, if applicable?

   ____

3. In which Southeast Minnesota County are your stakeholder interests primarily focused? [please check ☑ one]

   ☐1 Rice
   ☐2 Goodhue
   ☐3 Wabasha
   ☐4 Steele
   ☐5 Dodge
   ☐6 Olmsted
   ☐7 Winona
   ☐8 Freeborn
   ☐9 Mower
   ☐10 Fillmore
   ☐11 Houston
   ☐12 All or multiple counties equally

Some Questions About Your Community:

1. Which best describes your community?

☐ 1 Community with population <5,000
☐ 2 Community with population 5,000-20,000
☐ 3 Community with population 20-000=50,000
☐ 4 Community with population >50,000

2. We are looking for community champions to help develop a system for integrating evidence-based community resources into routine primary care in Southeast Minnesota. Would you be interested in learning more or partnering with us in this effort in your community? [please check ☑ one]

☐ 1 No, not at this time
☐ 2 Yes, my contact information is: (specify name and contact)

____

Thank you for completing this survey!

Community Stakeholder Semi-structured Interview Guide: The CDSMP Implementation Project

INTRODUCTION:

Thank you for taking the time to talk with me today. I am part of a regional collaborative seeking to create linkages between healthcare providers and effective resources in the community. As someone who has a potential stake in the health and wellness of people in Southeast Minnesota, you have special insight into factors that will help our team succeed. We are very interested in understanding your perspective.

In this interview, I will be asking you questions about your thoughts and experiences related to a community-based chronic disease management program. I will also ask you for your opinions about factors that may promote or inhibit its use and benefit in Southeast Minnesota. I want to assure you that your participation is voluntary and that you can choose to stop at any time. If a question makes you uncomfortable or you do not want to answer, that is fine; you do not have to. To respect your privacy, all of your responses will be held in confidence and no one will be able to connect you to any of the things you say.

You should feel open to express your thoughts and experiences honestly and to ask for clarification if any questions seem confusing.

Okay, do you have any questions before we get started?

WARM-UP CHAT (unstructured):

Okay, great. So, first I'd just like to learn a little bit about you.

1. Tell me about yourself.

    Potential probes

    a. Please tell me about your professional role (if applicable)
    b. How long have you been in that role?
    c. What are your responsibilities in your current role?
    d. Have you participated in any research studies before?

Transition: Okay, great. Now I would like to ask you more specific questions about an evidence-based health promotion program known as the Chronic Disease Self-Management Program.

2. How familiar are you with this program?
    a. If familiar consider the following question
        i. Tell me about your experience with the program.
    b. If not familiar review the following description with the participant.

THE CDSMP:

The Chronic Disease Self-Management Program is an effective program for improving the health and wellness of individuals. It is often offered in community settings and is also known as the “Living Well with Chronic Conditions” program. The program is for any adult with a chronic disease. A chronic disease can be thought of as any ongoing mental or physical health condition. Examples are things like depression and anxiety, diabetes, cancer, arthritis, and asthma. Participants in the program meet in groups that are led by a pair of trained leaders who also have at least one chronic condition. The participants meet once per week for 2 ½ hours for 6 weeks. The program provides education and support for self-management strategies like healthy eating and exercise, managing medications, dealing with emotions, communicating with others, making decisions, and understanding the mind-body connection. The program has been tested in many randomized trials and stakeholders such as the Centers for Disease Control, the Agency for Healthcare Research and Quality, and the Department of Health all recommend its use. I am interested in knowing a little bit about your perception of the potential value of this program.

Potential Probes

- How has the program impacted you, your practice, or your patients? What other community based health promotion initiatives are you familiar with or have you been a part of??

Transition: Now that we have discussed the Chronic Disease Self-Management Program (also known as Living Well with Chronic Conditions) in general, perhaps we could talk more specifically about the role of this program in the work of routine clinical practice.

BARRIERS/FACILITATORS TO IMPLEMENTATION:

3. Given your role, knowledge and experience what are some issues we will have to consider when trying to get the CDSMP integrated into routine clinical practice

Potential Probes

- Tell me about your current referral process for the CDSMP or similar programs.
- How could we best support you, your patients/clients/constituents in implementing this community based health promotion program?
- How do clinicians in your clinic/health system find out about community based programs like the CDSMP?
- If CDSMP referrals were to be implemented in your clinic/health system/service area what are some things that would need to be considered?
- If you or someone like you were to participate in a CDSMP what would make you more or less likely to participate?

- As a potential provider of CDSMP workshops, what would facilitate or impede your ability to provide these programs?
- What resources does your organization have available to support this sort of initiative or what type of resources would your organization require?
-
- As a person who may potentially refer people to the CDSMP, what factors make it more or less likely for you to refer them?
- What do you think the best infrastructure or system would look like for increasing clinician referrals to programs like the CDSMP?

Transition: This has all been very helpful. Before we close, I have one final question.

4.Is there anyone else we should talk to about these programs?

Potential Probe

- In your role, who would be impacted by these programs?

Closing: I think that's all I need unless you have any other comments or questions. Okay, thank you again for taking the time to chat with me about this.

Clinician Interviews	Stakeholder Interviews
Predisposing Factors	
C101: "Yeah, I think that's, that's where we need some basic information on what the various programs out there can provide."<br><br>C103: "So for instance somebody who doesn't necessarily trust medications or even though they may really need medications based on my recommendations wanna avoid meds, avoid drugs, find a more holistic approach to it. And I find that my knowledge there is fairly limited. So the yoga, the tie-chi and the truly making dietary changes and, and what not I think there's a need there."<br><br>C103: "Unfortunately only a lot of those venues may not have oversight to make sure that the discussions are correct. Certainly whatever the person is feeling is correct but that they're sharing correct information about best practices. I find a lot of people coming in with, with ideas that may not be evidence based or may even be harmful to their health."	S3: "a lot of research has been done and they actually did a really large like 1 year follow up study. So the workshops only 6 weeks long but they followed up with participants a year later and they found still improvements in like 10 different areas. And I probably can't ramble off all 10 but … self-reported health, depression, self-management skills, sleep, healthcare or doctor visits, reduce number of doctor visits, oh and there's several others."<br><br>S5: "There is no real knowledge among clinicians about the benefit of this. That is the biggest challenge. I think the perception is that it is probably a nice program but is not going to address critical issues in healthcare."<br><br>S14: "I always say when I'm trying to work with a clinic and say, you know, you can't just have the doctor know about the program; you have to have the front desk . . . . . . somebody . . . . . . whoever's answering the phone needs to know it."
Reinforcing Factors	
C108: "Well I think, in general, there is a medicine culture that is going to be a barrier. I think physicians are reluctant to delegate any sort of responsibility for care to you have a hard enough time getting them to delegate the responsibility to people who sit in the same office as them. Having them further delegate that to people who they don't know at all outside of the healthcare system is going to be a challenge"<br><br>C102: "I guess the, the other thing I might think about and, and this all about again perspective and perception. And that is that it might be seen as competing with management of the care of a patient to a provider. People who understand the changes	S4: "But um you know you you can't make people believe in something until either they have to do it or you've convinced them. Is that we just have to keep repeating it and repeating it and repeating it until it starts to become an integral part ah of how we see things and how clinicians think about treating their patients. That it's not just about what medication you should take. You know it's something we have to create an understanding that they should think they should have to have"<br><br>S3: "But, working - having a physician champion, like [edited] and being able to build some of that credibility that he brings to the table and that the collaboration brings to the

in medicine that are needed probably would be less concerned about that. But I could certainly see that being an issue that might come up."<br><br>C105: "But if there are 5 or 6 programs out in the community that are successful then all of a sudden you're thinking ok there's this community, there's this package of community resources out there and, and will, will end up using it more because it becomes a more common thing."	table, has really overcome that barrier pretty well. I mean when we get in and talk to different provider groups the way we're able to do it now the buy-in usually isn't too hard. But when for years, when it was just me trying to do this it was a whole different story."<br><br>S1: "Our mental model must change from thinking of the clinic as where chronic disease is managed to recognizing it is managed in the kitchen, bathroom, etc. We don't have an ecosystem where we can celebrate the flourishing of the patient due to a correct referral to another clinician or agency."
Enabling Factors	
C101: "So I think there's I think there's potential but you know *boy* I would look at --- you know some of the providers are gonna say, "I don't want another button to click or another orders" (laughing) but it's really the easiest way to to connect people with these programs I think."<br><br>C111: "I was given a very brief education on what it was. You know, it was basically like, send us referrals; we'll help these people. I mean, it was very brief, and so I was like well, one, I don't know you know, if I usually recommend something, I can usually give some details about it when people ask questions. So what does it mean? What do we talk about? I had zero answers for any of those (laughs), so I felt like it was hard for me to really strongly recommend it cuz I didn't know that much about it."<br><br>C106: "You know, we might have a 20-minute appointment, and 40 minutes of things that we need to go over medically with the patient. So I think it's very, very hard for providers to be able to bring that up; but, like with the nursing staff or even our front-desk staff, having someone that's very knowledgeable	S4: "I think doctors are a lost cause (chuckles)…and and and that's not an insult to them it's just I'm a patient and and you know when I have my own family physician who I've seen for over 20 years, tell me when I come in to see him and I raise a question and he says, you know I'm really sorry but I can't talk to you about that today cause I've only been slated for 20 minutes with you and so you're gonna have to schedule another appointment and come back to talk to me about that. I you know that says it all"<br><br>S3: "But I think the biggest barrier is that the - if we're talking specifically about physicians - that they're already overwhelmed in their appointment time with their patients. And host of other topics and things they're supposed to be thinking about and referring to and talking about."<br><br>S9: "And I'm you know thinking about the, the financial I'm afraid that it's going to end before we're able to really show people how beneficial the program is. Does that make sense? Ye, so it's like I'm I feel like I'm ti-ticking time bomb here (interviewer chuckles) that ok I'm gonna refer these people, I've got

within the clinic that we can say, hey, this patient, I think, is a really good candidate for this; can you go in and talk to them about this?”	more to refer, I’ve got more to talk to about it and because of funding, people are going to you know not continue this program. And I think it’s *really* important to have an outside source and to work together.”
Administrative Issues	
C101: “And if it in my opinion if we had a button that (taps tables) that list it you know community, community agencies or (background noise). Gave us a list and we could hover on it and have a brief explanation of what it was. And we could click it, it went to the desk and they made the contact. I think that would work the best. (131-142)”<br><br>C108: “I get frustrated that I am expected to diagnose and help someone manage a condition and, remember, I’m really only trained to do the diagnosis part. And I can talk through the treatment options with them, but I mean those are I can’t really go much deeper than saying—you should take this medication at this dose.”<br><br>C102: “And, and, and they feel like there that not only their patient is getting something out of it, which is clearly the, the main goal. But that there, that they’re getting something out of it as well that’s gonna make it better for them to manage maybe to be able to hit targets for, for care management goals related to quality outcomes and service outcomes and safety outcomes and so on. So that it so, so this is seen as a beneficial from those ends.”	S10: “We don’t have a a process like where it goes through their medical record because we feel that we don’t need to do that, and it’s really not could be a HIPPA you know what I mean? Kike cause it’s not medically prescribed. Now could that be changed? Absolutely… Unfortunately all of our referrals are, are legitimate referrals or whatever you call em are are done through the medical record. Could we do it that way, sure.”<br><br>S5: “The only way clinical leadership will take interest in the program is if it could free up capacity by offloading some of the activities that nurses are currently doing (paraphrase).”<br><br>S5: “So the practice level really, I think what would get their attention. One include this is a somehow this leads to better performance measures as the practice measures it or lower cost.”
Policy Issues	
C102: “Part of the issue may be you know there, there are so many things coming at, at us in healthcare these days. including expectations and requirements about different ways to care for patients. About things that we have to meet for metrics and measures. And ne of the one of the barrier issues might just be a volume overload issue.	S3: “It’s, it’s probably kind of our biggest most complicated - you know area workgroup that we have. Because there’s a lot of state politics involved too. And so CDSMPs implemented not only statewide but nationally but it’s, it’s really focused on by state.”<br><br>S4: “Nationally when all of these evidence

Is this another thing; is this another thing I have to get 'em connected to?"<br><br>C104: "And the truth is that there times I tell you that you know I'm, I'm missing even I'm not gonna say more critical things but – but things that I *measure* on. I measure on how many mammograms we do, I measure on what my patients have influenza. Vaccination and I'm even with the technology time wise and attention wise and when you're doing so many things you missed that. So (sighs) is there, is there an automatic way to do this?"<br><br>C109: "But you need a room. You need a population of patients. You need instructors who are giving of their time, either on a volunteer basis or are paid. You know, so who's going to finance all of that stuff? And you know, it's one thing to provide, you know, conventional established services—like a doctor meets with a patient, and we can charge them X amount of money. But if these things aren't reimbursed by any entities, then it's harder."	based programs were um funded by the older Americans Act, that thought was that we were gonna educate doctors and they were gonna be telling patients about these programs and classes but that's not realistic. You know their time ah is so prescribed in many of the clinics across the country that that's just not a feasible approach. So I think our best bet is the next step down of people who work with the patients hands on."<br><br>S3: "So a portion of the workshops that go on are foundered, funded under older Americans act title 3D funding. Which is that funding has to be used specifically for evidence based health promotion programs but they also have to be geared towards seniors. So that's a limitation of that. And then another portion of the workshops going on, the organizations are just taking it on as part of their integrated part of what they do as their organization. So right now it's a combination of that and that's one thing, kind of a big thing that we have to figure out going forward is, are we able to continue to do this free of charge?"
